# Enhanced removal of bisphenols using immobilized TiO_2_ in photocatalytically assisted hydrodynamic cavitation

**DOI:** 10.1016/j.ultsonch.2026.107830

**Published:** 2026-03-28

**Authors:** Andraž Šuligoj, Mojca Zupanc, Jurij Gostiša, Pia Hrovat, Ester Heath, Nataša Novak Tušar, Urška Lavrenčič Štangar

**Affiliations:** a Faculty of Chemistry and Chemical Technology, University of Ljubljana, Večna pot 113, SI-1000 Ljubljana, Slovenia; b Faculty of Mechanical Engineering, University of Ljubljana, Aškerčeva cesta 6, SI-1000 Ljubljana, Slovenia; c Jožef Stefan Institute, Jamova cesta 39, SI-1000 Ljubljana, Slovenia; d International Postgraduate School Jožef Stefan, Jamova cesta 39, SI-1000 Ljubljana, Slovenia; e National Institute of Chemistry, Hajdrihova 19, SI-1000 Ljubljana, Slovenia; f University of Nova Gorica, Vipavska 13, SI-5000 Nova Gorica, Slovenia

**Keywords:** Hydrodynamic cavitation, Photocatalysis, Immobilized TiO_2_, Quaternary treatment, Bisphenols

## Abstract

Bisphenols are widespread in the environment and can cause harm to aquatic and terrestrial life by acting as an endocrine disruptor, affecting reproduction, growth, and development. Hydrodynamic cavitation and photocatalysis can both be used to remove bisphenols from aquatic bodies, yet the reports on the combination of the two in a single system are scarce. Herein, we studied the removal of five bisphenols from tap water (TW) and real wastewater (WW) effluent at two environmentally relevant concentration levels, 200 ng/L and 1000 ng/L, by means of hydrodynamic cavitation, both independently and in combination with photocatalysis, using a TiO_2_-SiO_2_ composite catalyst immobilized on Al_2_O_3_ monoliths. For tap water, inside the short treatment times (15 min or 30 min) the efficiency of removal reached up to 100% for tetramethyl bisphenol F (TMBPF) while only 15% for bisphenol S (BPS). Interestingly, the average total amount of BPAs removed across all treatment combinations was almost the same in case of TW (58.5 ng) and WW (60.4 ng). The current study shows an important advancement in the practical applicability of the two methods for treating polluted water bodies which is applicable to the upcoming implementation of the quaternary treatment in WW treatment plants.

## Graphical abstract

The graphic abstract is optional. It should be a representative of your entire work and not be a duplicate of a graphic already used in the manuscript. Color structures, graphical images, photographs, or reaction schemes are typically good choices, which may be chosen as cover picture.

**Figure:**
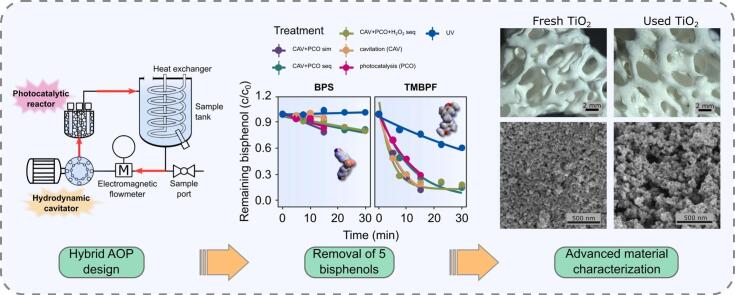

## 1 Introduction

An estimated 80% of the world’s population faces high threats to water security [1]. While large portion of drinking water comes from groundwater its quality is inevitably linked as 85% of groundwater withdrawals are sourced from surface water capture and reduced evapotranspiration, and the remaining 15% from aquifer depletion [2]. It is also projected that production of wastewater will double by 2050 [3]. Yet, wastewater reuse introduces challenges, as treated effluents are a primary source of micropollutants [4] that may accumulate in crops and enter the food chain. To mitigate these risks, efficient removal of micropollutants is critical.

**Fig. 1:**
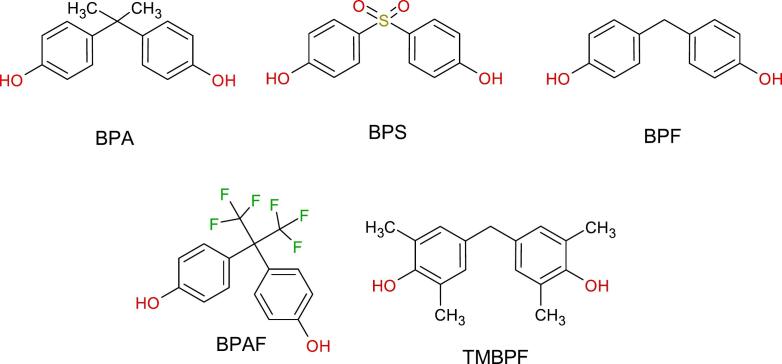
BPA analogue structures. The chemical structures of BPA and four new, commonly used BPA alternatives.

Among the many classes of micropollutants, bisphenols are of particular concern. These industrial chemicals share a common structure of two phenol units connected by a bridging group [5], [6]. While bisphenol A (BPA) is one of the three benchmark pollutants for endocrine disruptors in the environment by the EU directive 2020/2184 [7], several structural analogues — such as bisphenol S (BPS), bisphenol F (BPF), bisphenol AF (BPAF), and the more recently introduced tetramethyl bisphenol F (TMBPF) (Fig. 1) — are increasingly detected in the environment. These compounds exhibit endocrine-disrupting properties similar to BPA [8], [9] and emerging evidence suggests that some analogues may pose even greater toxicological and teratogenic risks, with potency following the order BPAF > TMBPF > BPS > BPA [10]. Additionally, their bioaccumulative potential is highly dependent on their hydrophobic nature [11] which varies greatly in the group of synthesised bisphenols.

Conventional treatment processes only partially remove micropollutants. Recognizing this gap, the European Parliament adopted Directive 2024/3019 on urban wastewater treatment, which requires member states to implement quaternary treatment for plants treating ≥ 150,000 population equivalents (PE) by 2045. For plants serving ≥ 10,000 PE in sensitive areas – such as drinking water sources, bathing waters, or shellfish production zones – quaternary treatment will also be required unless risk assessments demonstrate negligible risk. The directive sets a target of ≥ 80% removal for selected micropollutants [12], [13].

Advanced Oxidation Processes (AOPs) are among the most promising technologies for quaternary treatment due to their ability to generate highly reactive species (e.g., ^•^OH) capable of non-selectively degrading persistent organic contaminants. AOPs can be integrated with biological, physical, or chemical treatment processes to enhance overall performance. However, they may be energy-intensive, require precise operational control, and incur costs related to reagents (e.g., H_2_O_2_, O_3_) and equipment maintenance (e.g., UV lamps) [14].

Combining different AOPs has been proposed as a strategy to overcome these limitations and achieve higher removal efficiencies. For example, integrating cavitation with photocatalysis has been reported to enhance contaminant degradation beyond the sum of the individual processes [15]. Cavitation — whether acoustic (AC) or hydrodynamic (HC) — generates localized extreme conditions (high temperature and pressure) that promote the formation of reactive radicals [16], [17]. When coupled with photocatalysis (e.g., TiO_2_ under UV irradiation) or addition of external oxidants (e.g., H_2_O_2_), this synergy accelerates the breakdown of target compounds, improves mineralization, and can reduce overall treatment time [18], [19]. Previous studies have demonstrated the effective degradation of phenolic pollutants, such as 4-chlorophenol, under combined cavitation-photocatalytic conditions, although challenges such as photocatalyst deactivation remain [23]. Also, the photocatalysts were used in suspended form, which limits application value of such a system. Even more critical is the unknown behaviour of such hybrid systems under conditions closer to real applications, i.e., at concentrations of pollutants orders of magnitude lower than what has been studied up to now. Environmental monitoring showed values of bisphenols in aquatic environments ranged from non-detectable to 0.763  µg/L in lake water [20]. At such low concentrations, issues of mass transfer as well as adsorption competition and illumination shielding become important.

In this study, we evaluate the combination of HC and immobilized TiO_2_-photocatalysis to enhance the removal of five bisphenols (BPA, BPS, BPF, BPAF, and TMBPF) at environmentally relevant concentrations (≤1 µg/L). Our work aims to provide insight into the synergistic effects of this combined process and assess its potential as a scalable quaternary treatment technology for micropollutant removal.

## 2 Experimental

### 2.1 Materials and chemicals

The following chemicals were used as purchased: BPAF (≥ 98%), BPF (≥ 98%), BPA (≥ 98%), BPS (≥ 98%), titanium(IV) isopropoxide (TTIP, 97%), and tetraethyl orthosilicate (TEOS, ≥ 99%) from Sigma-Aldrich (St. Louis, USA) and TMBPF (≥ 98%) from Tokyo Chemical Industry (Tokyo, Japan). Isotopically labelled BPF (^13^C_12_-BPF) (≥ 95%) and BPS (^13^C_12_-BPS) (≥ 95%) were purchased from CanSyn Chem. Corp. (Toronto, Canada), BPA-d_16_ (≥ 98%) from Sigma-Aldrich (St. Louis, ZDA). Acetonitrile (AcN, ≥ 99.8%), ethyl acetate (EtAc, ≥ 99.5%) and methanol (MeOH, ≥ 99.8%) were purchased from J. T. Baker (Deventer, The Netherlands) while absolute ethanol was purchased from Gram-Mol (Zagreb, Croatia). Hydrochloric acid (HCl, 37%) and formic acid (FA, ≥ 98%) were provided by Sigma Aldrich (St. Louis, USA) while perchloric acid (HClO_4_, 60%) was obtained from Merck. Aeroxide TiO_2_-P25 was purchased from Evonik Industries AG (Essen, Germany). The derivatising agent N-metil-n-(trimethylsilyl)-trifluoroacetamide (MSTFA, ≥ 99%) and catalyst anhydrous pyridine (99.8%) were provided by Sigma-Aldrich (Schnelldorf, Switzerland and Steinheim, Germany). Hydrogen peroxide (H_2_O_2_, 30%) was purchased from Belinka Perkemija (Ljubljana, Slovenia). Ultrapure water (MQ, specific resistance 18,2 MΩ/cm at 25 °C) was obtained using the MilliQ-water purification system (Millipore Merck Direct-Q^TM^). Ceramic monoliths consisting of Al_2_O_3_ (10 PPI porosity, ϕ = 72 mm, h = 26 mm) were purchased from Lanik (Boskovice, Czechia).

Wastewater was acquired from the Domžale-Kamnik WWTP on 19. 2. 2024, 20. 2. 2024 and 21. 2. 2024. Samples were taken at 8:00 from the same Sequencing Batch Reactor (SBR) 10  min after the start of the third phase before the release of water into the nearby river Kamniška Bistrica. They were used as is, unfiltered. Tap water was acquired from the municipal waterline of the city of Ljubljana and its parameters are shown in Table S1 in the supporting information file.

The LogP parameter was predicted by the Chemsketch 2024.1.0 software.

### 2.2 Preparation of the photocatalyst

The photocatalyst was prepared by immobilising TiO_2_ nanoparticles on monoliths composed of corundum (Al_2_O_3_), according to the method described previously [21].

A sol-suspension of commercially available TiO_2_ NPs was prepared by mixing 30 mL TTIP and 5 mL of ethanol. A 2 mL perchloric acid solution in 90 mL water was also prepared. After stirring the TTIP solution for one hour, the perchloric acid solution was added dropwise, serving as a hydrolysis catalyst while stabilising the sol. The hydrolysis was exothermic, and the prepared suspension was stirred under reflux for 48  h [21].

A silicate sol was prepared separately to serve as the binding agent for immobilising the catalyst by mixing 26.04 mL of TEOS with 14 mL of MilliQ water. While stirring, 91 μL of HCl was added [22].

The final sol-suspension was prepared by mixing 7.2 mL of silicate sol, SiO_2_ colloidal solution (Levasil 200/30%) and 96 mL of ethanol to 50.4 mL of TiO_2_ sol and stirred for 30 min. To this, 19.2 g of powdered TiO_2_ P-25 was added. The suspension was homogenised in an ultrasonic bath for 10 min. The suspension was stirred overnight before diluting with 165 mL of ethanol. With the solution remaining in the ultrasonic bath, the monoliths were dip-coated for 10 s at a 10 cm/min withdrawal speed. Afterwards, the monoliths were thermally treated at 150 °C for 1 h. The coating and drying process was repeated three times.

### 2.3 Experimental test rig

The two main components of the reactor system are the photocatalytic reactor (PCO) and the hydrodynamic cavitation generator (CAV), which also functions as a pump. The CAV is essentially a poorly designed centrifugal pump intended primarily to induce cavitation. It is driven by a Kollmorgen AKM42J servo motor with a rated power of 1.5 kW. The rotational speed was set to 3500 rpm in the pumping regime and 5500 rpm in the cavitation regime. The PCO contained three centrally positioned Philips TL 4 W G5 BLB Blacklight lamps (UVA Hg, λ_max_ = 365 nm). Three Al_2_O_3_ monoliths (26 × 72 mm^2^, porosity 10 PPI, Lanik, Czechia) were stacked in the reactor (0.230 L), providing a total geometric area of approximately 4.05 m^2^ [23]. This resulted in a total photocatalyst mass of ∼ 40 g in the reactor.

**Fig. 2:**
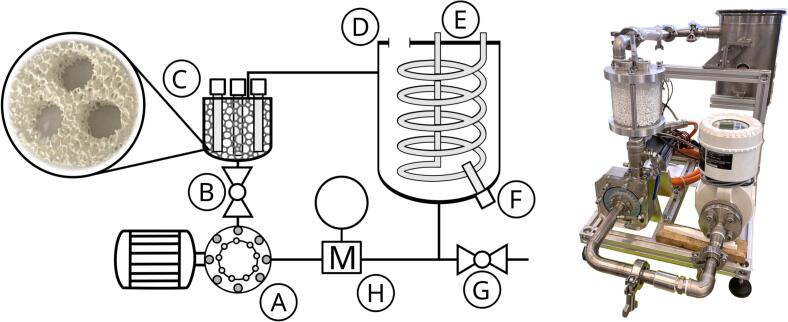
Experimental rig with the key components: A − cavitation generator, B – throttling valve, C – photocatalytic reactor with 3 monolith inserts, D – sample tank, E – cooling coil, F – temperature probe, G – sampling port, H – electromagnetic flow meter.

PCO and CAV were installed in a closed-circuit experimental rig, schematically shown in Fig. 2, where sample treatment can be performed in a batch configuration. The sample batch is stored in a 5 L sample tank (Fig. 2 − D) with a cooling coil (Fig. 2 − E) connected to a laboratory cooling device. From the tank, the sample flows through the electromagnetic flowmeter to the CAV (Fig. 2 − A) and then continues to the photocatalytic reactor. A throttling valve is installed between these two components, allowing adjustment of the flow rate (Fig. 2 − B). During the tests, the flow rate was maintained at 6 L/min and the sample from the PCO is re-entering the tank (E).

Seven different regimes were performed using tap water (TW) and wastewater (WW) (Table 1): (a) adsorption of bisphenols on the photocatalyst in the dark, (b) 15 min of PCO, (c) 15 min of CAV, (d) 15 min of simultaneous PCO and CAV (simultaneous), (e) 15 min of CAV followed immediately by 15 min of PCO (sequential), (f) 15 min of CAV followed immediately by 15 min of PCO with the addition of H_2_O_2_ (sequential), and (g) 30 min of UV irradiation (with the photocatalyst removed from the photocatalytic reactor) (photolysis). 15 min of reaction time resulted in 18 passes while 30 min in 36 passes. For the TW experiments, all tests were conducted using a single batch of monoliths coated with the photocatalyst. The monoliths were replaced, and the first series of WW experiments (a)–(g) was performed. Afterwards, the monoliths were replaced, and the second series of WW experiments (a)–(g) was conducted. Experiments were performed in duplicates, and the results represent the mean of the two experiments. The results did not deviate for more than 5%.

**Table 1:** Investigated experimental conditions. Total system volume was 5 L and volumetric flow rate was set at 6 L for each experiment.

**Treatment**	**Experiment label**	**Rotational speed (rpm)**	**Treatment time (min)**	**P_CAV_ (W)**	**P_PCO_ (W)**	**Bisphenol conc. (ng/L)**	**ΔT (*t*_end_ − *t*_start_) (°C)**	**Bisphenol conc. (ng/L)**	**ΔT (*t*_end_ − *t*_start_) (°C)**
Adsorption	AD	3500	30	270	−	200	−	1000	−
Photocatalysis	PCO	3500	15	270	12		16.3		17.4
Cavitation	CAV	5500	15	1100	−		25		27.5
Photolysis	UV	3500	30	270	12		16		16.2
Simultaneous cavitation + photocatalysis	sim CAV+PCO	5500	15	1100	12		25.6		27.8
Sequential cavitation/photocatalysis	seq CAV+PCO	5500/3500	15 + 15	1100/270	12		25.7/18.0		27/17.5
H_2_O_2_ + Sequential cavitation/photocatalysis	seq CAV+PCO+H_2_O_2_	5500/3500	15 + 15	1100/270	12		25.7/18.3		27/18.7

Samples were spiked with BPAF, BPF, BPA, BPS and TMBPF at 200 ng/L each in tap water and 1000 ng/L in wastewater. The higher concentration in wastewater was chosen due to expected matrix effects in real samples. In the case of 15 min experiments samples were collected at 0, 5, 10 and 15 min, whereas in the case of 30 min experiments samples were collected at 0, 7.5, 15, 22.5 and 30 min. The reaction system was flushed with tap water for 5 min between individual experiments, using UVA lamps at 5500 rpm to clean the system.

### 2.4 Characterisation techniques

Combined thermal gravimetric analysis (TGA) and dynamic scanning calorimetry (DSC) was carried out using a Mettler Toledo TGA/DSC 1 under air flow with a 5 K/min heating rate. Approximately 10 mg of sample was placed in a 150 µL platinum crucible on the pan of the microbalance and heated from room temperature to 700 °C.

Diffuse reflectance (DR) spectra of the solids were recorded on a Lambda 650 UV–Vis spectrophotometer (Perkin Elmer, USA), equipped with a Praying Mantis accessory (Harrick). The scan speed was 480  nm/min and the slit was set to 2  nm. Spectralon® was used for background correction. The attenuated total reflectance FT-IR/ATR spectra were recorded on a Perkin Elmer spectrometer (Spectrum 100, USA) using MIR TGS detector. Spectra were recorded from 4000 to 400 cm^−1^ with the resolution of 2  cm^−1^ using diamond crystal in a horizontal position.

Scanning electron microscope SUPRA 35 VP (Carl Zeiss, Germany) field-emission scanning electron microscope operating at 2  kV and using a 30  µm aperture was used to examine the surface morphology of the synthesized catalysts and to observe the textural features of the materials.

X-ray powder diffraction (XRD) patterns were recorded on an X’Pert PRO (PANalytical, the Netherlands) high-resolution diffractometer using CuKα_1_ radiation (λ = 1.5406 Å).

Nitrogen physisorption isotherms were measured on a SYNC 220A apparatus (3P, Germany) recording at 77 K. The samples were outgassed at 120 °C for 2  h.

### 2.5 Analytical methods

After sampling, 10 µL of a mixed standard solution containing ^13^C_12_ - BPF, ^13^C_12_ - BPS, and BPA - d_16_ (0.5  mg/L) was added to each 50  mL drinking water sample, while 50  µL of the same solution was added to each 50  mL wastewater sample. The bottles were sealed and stored until analysis, later the same day. Before preparation, the wastewater samples were filtered through GF/C filters (1.2  µm; Whatman, USA). Samples were first acidified to pH 2 with 100 µL of HCl (37%) and then extracted using solid phase extraction (Oasis HLB Prime cartridges, 60 mg, 3 mL; Waters, Massachusetts, USA) on a vacuum manifold (Vacuum Manifold, Agilent Technologies, USA). The samples were loaded onto the cartridges at 3 mL/mL. The cartridges were then rinsed with 10% MeOH in MQ water and vacuum-dried at −1.33 kPa for 45 min. The analytes were eluted using 1.8 mL of 5% FA in EtAc into 2 mL vials and dried under N_2_ at 40 °C.

For derivatisation, 50 µL of pyridine and 50 µL of MSTFA were added to the samples, mixed, and derivatised for 2 min in a microwave oven (800 W). After derivatisation, the vials were cooled to room temperature. The contents were transferred to a glass insert ready for analysis by GC–MS.

### 2.6 Instrumental analysis

All samples were analysed using a gas chromatograph (7890B, Agilent) with a mass-selective detector (GC–MS, 5977A, Agilent). Separation was achieved using a DB-5 MS capillary column (26.872 m × 0.25 mm × 0.25 µm, Agilent) with helium as the carrier gas at a 0.7 mL/min flow rate. Injection volume was 1 µL in splitless mode at 250 °C. The oven temperature was programmed from 120 °C to 250 °C at a rate of 20 °C/min (6.0 min), followed by a ramp to 300 °C at 10 °C/min (3.0 min). The solvent delay was 6.5 min. The total analysis time was 20.5 min. The electron impact (EI) energy was 70 eV. The target compounds were identified and quantified using selected ion monitoring (SIM mode). The monitored SIM ions and retention times (RTs) for the derivatised BPs and internal standards are presented in Table 2. Data were processed using MassHunter software (Agilent Technologies).

**Table 2:** Bisphenol analytes via GC–MS analysis.

**Bisphenol**	**Retention time** **[min]**	**Quantifier ion in bold and qualifier ion [*m*/*z*]**	
BPAF	7.58	**411**	480
BPF	8.70	**344**	179
BPA	9.23	**357**	372
TMBPF	12.11	400	**385**
BPS	15.34	379	**394**
^13^C_12_-BPF	8.70	341	**356**
BPA-d_16_	9.15	**368**	386
^13^C_12_-BPS	15.33	391	**406**

The TOC of aqueous samples was determined using Shimadzu TOC-L 5000A Analyzer with ASI-L feeder. Prior to analysis, samples were filtered through 0.2 μm Nylon filter (Chromafil).

### 2.7 Method validation

The limit of quantification (LOQ) in tap water was determined as the lowest value meeting a signal-to-noise ratio of 3:1. An eight-point calibration curve was prepared in triplicates over the range from LOQ to 500 ng/L. Method and instrument repeatability and accuracy were determined at two points on the calibration curve. Three replicates were prepared for each calibration point, and one sample was injected four times. The extraction efficiency, drying efficiency, and matrix effect were also evaluated from three replicates for each point. The matrix effect was assessed by comparing peak areas of standards in the samples with those in pure solvent.

For the validation in wastewater, the LOQ was estimated as the lowest value meeting a signal-to-noise ratio of 3:1. A ten-point calibration curve was prepared in pure solvent in triplicate over the range from LOQ to 2000 ng/L. Method, instrument repeatability, and accuracy were determined at two points of the calibration curve, with three replicates for each point, and one of the samples was injected four times. The filtration efficiency, extraction efficiency, drying efficiency, and matrix effect were determined at two points of the calibration curve, with three replicates for each point.

### 2.8 Removal efficiencies

The removal efficiencies of the five bisphenols were analysed according to the commonly used Langmuir-Hinselwood model [24]. In this model, the decay rate is *r*, *C* is the concentration of the organic molecule, t is the time of light irradiation or cavitation, *k* is the decay constant, and *K* is the L-H equilibrium constant (empirically determined), equation (1).

(1) $$r=-\frac{\mathrm{dC}}{\mathrm{dt}}=-\frac{k_{r}K_{e}C}{1+K_{e}C}$$

where *C* represents the concentration in solution of the molecule being degraded (mg/L), *k*_r_ is the reaction rate constant (mg L^−1^ min^−1^) and *K*_e_ is the equilibrium constant for the adsorption of the molecule on the catalyst surface at the reaction temperature (L/mg). The term *k*_r_ *K*_e_ is globally evaluated as an apparent rate constant (*k*_app_; min^−1^) [1]. Thus, Eq. (1) can be rewritten as:

(2) $$-\frac{\mathrm{dC}}{\mathrm{dt}}=-\frac{k_{\mathrm{app}}C}{1+K_{e}C}$$

Because in our case the concentration of bisphenols is very low (*K*_e_*C* ≪ 1), Eq. (2) can be rewritten as a classical first-order reaction:

(3) $$r=-\frac{\mathrm{dC}}{\mathrm{dt}}=-k_{\mathrm{app}}C$$

After integration, we obtain the non-linear model equation:

(4) $$C_{t}=C_{0}e^{-kt}$$

Quantum chemical calculations were performed using the ORCA version 6.1.1. Molecular structures of the five bisphenols were obtained from the PubChem database as 3D conformers and subsequently subjected to full geometry optimization at the B3LYP/6-31G* level of theory. No symmetry constraints were imposed during optimization. Geometry convergence was confirmed for all structures.

Single-point energy calculations were performed on the optimized geometries at the B3LYP/6-311+G** level of theory, which provides an improved description of electronic properties through the inclusion of diffuse and polarization functions [25]. Molecular orbital energies were obtained from the converged single-point wavefunctions. The energies of the highest occupied molecular orbital (HOMO) and lowest unoccupied molecular orbital (LUMO) were extracted from the orbital energy output. Ionization potentials (IP) and electron affinities (EA) were estimated from the HOMO and LUMO energies, respectively, according to Koopmans' theorem.

## 3 Results

Before the materials underwent catalytic testing, they were characterized using diffraction, spectroscopic, and thermal analyses. After use in the reactor systems for bisphenol removal, they were characterized again to provide insights into possible structural and chemical changes and their suitability for long-term applications. Therefore, we first discuss the removal efficiencies achieved with different combinations of AOPs, followed by the physico-chemical characteristics of the fresh and used materials.

### 3.1 Removal efficiencies in tap water

**Fig. 3:**
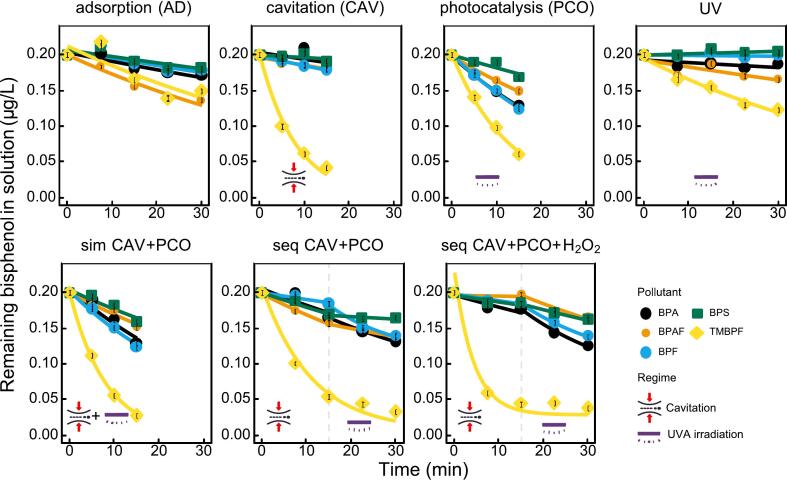
Removal of five bisphenols (BPA, BPAF, BPF, BPS, TMBPF) in tap water at 200 ng/L under different treatment conditions: adsorption (AD), cavitation (CAV), photocatalysis (PCO), UV irradiation, and combined processes (CAV + PCO simultaneously and sequentially, with/without H_2_O_2_). The solid lines represent data fitted to the non-linear form of the first-order reaction rate (Eq. (3). The dashed lines represent regime change, in case of sequential hybrid regimes.

Fig. 3 shows that the degree of adsorption onto the catalyst was low for all bisphenols (only about 5–15% after 30 min), except for BPAF and TMBPF, which were adsorbed at 32% and 25%, respectively. This suggests limited physisorption on the catalyst surface or HC reactor walls. The higher adsorption of BPAF and TMBPF could be explained by their greater hydrophobicity (logP(BPAF) = 4.44 and logP(TMBPF) = 4.57) compared to the other compounds (from 1.83 to 3.43). In the cavitation regime, TMBPF showed the highest degradation (almost 80% removal at 15 min), while the other bisphenols remained nearly unchanged. For these bisphenols, on average, CAV showed removal kinetics comparable to adsorption (*k* = 0.0069 ± 0.005 min⁻^1^). This suggests that TMBPF is better removed with cavitation than with PCO. In the PCO regime, TMBPF also degraded fastest (approximately 70% removal at 15 min), while BPF, BPA, and BPAF showed moderate degradation, and BPS was least reactive (barely decreased). The UV regime showed low direct photolysis except for TMBPF (still modest), which, compared to the PCO process, demonstrates that removal in PCO truly is a photocatalytic process rather than direct UV-driven decomposition.

Regarding the hybrid processes (bottom panels in Fig. 3), the simultaneous configuration showed faster kinetics only in the case of TMBPF and BPS, meaning that these two bisphenols were removed faster in the hybrid process than with CAV or PCO alone. However, no synergy could be claimed even for these two bisphenols. Additionally, when the processes were put in sequential configuration, the final removal degree was similar to CAV alone for TMBPF and to PCO alone for the other four bisphenols. In sequential configuration a second stage of bisphenols removal (15 min ≤ *t* ≤ 30 min) can be seen (Fig. 3), which could be again fitted to the apparent first order kinetics (Eq. (3). The existence of a clear second stage is due to the fact that the sites for photocatalytic generation of ROS are spatially and temporally distinct from the source of CAV. Comparing the PCO stage alone and after the CAV stage (in seq CAV + PCO) shows that for all pollutants, kinetics is slower for the latter case. The elevated temperature (2229 °C during 15 min of CAV, Table 1) was reported to be insignificant to the rate constants of BPA removal during photocatalysis both when TiO_2_ was immobilized onto similar geometrically formed foams [26] or when used in powder form [27]. The other possible culprit could be the presence of intermediates which could slow down the further degradation of the parent bisphenols in the PCO stage.

We then added additional oxidant in form of H_2_O_2_ in the sequential configuration. These experiments showed the strongest overall removal (TMBPF nearly fully degraded, others significantly reduced). This implies that H_2_O_2_ results in larger ROS production. Yet the kinetics of the first stage (CAV) was practically the same as CAV stage alone (without H_2_O_2_) which implies hydrogen peroxide does not benefit the production of ·OH within the process of hydrodynamic cavitation. Also, the kinetics of PCO removal was significantly slower in this case than in PCO stage alone, implying on the same mechanism of removal with PCO as in the seq CAV + PCO configuration.

### 3.2 Removal efficiencies in wastewater

**Fig. 4:**
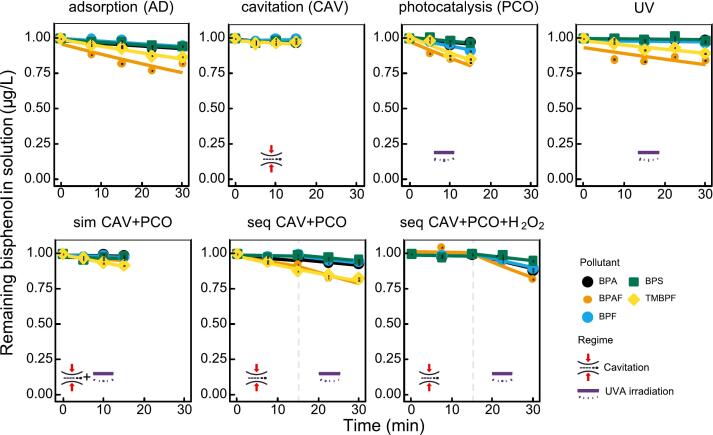
Removal of bisphenols from wastewater. The solid lines represent data fitted to the non-linear form of the first-order reaction rate (Eq. (3). The dashed lines represent regime change, in case of sequential hybrid regimes.

Expectedly, across all treatments, removal efficiencies in WW were significantly lower than in TW (Fig. 4). After 30 min, none of the processes achieved > 25% degradation (vs. > 90% removal of TMBPF in TW under CAV + PCO). The results imply matrix effects — scavenging of reactive species, light attenuation, and possibly competitive adsorption. Interestingly, bisphenols showed measurable adsorption to the photocatalyst, especially BPAF and TMBPF (around ∼ 20%). This trend was the same as in TW. Conversely, cavitation regime alone showed almost no degradation. This can be a consequence of mainly two factors: (i) natural organic matter (NOM) scavenges radicals produced during cavitation, (ii) bisphenols get adsorbed to NOM and thus have less contact with the cavitation bubbles. Since photocatalyst showed reasonable adsorption as well as detectable removal of bisphenols in WW this implies that availability of ROS is the main culprit. The results are very similar to our previous study using serrated pin HC in case of BPS, BPF and BPA removals, whereas 49% removal was achieved for BPAF [28]. Following this trend, it is seen that PCO showed removals limited to ∼ 10–20% consistently higher than CAV. Both parallel and simultaneous hybrid configurations slightly outperformed single processes, but enhancement was much weaker than in TW. Parallel configuration showed only ∼ 10–15% removal implying that cavitation did not significantly enhance mass transfer or ROS generation in this matrix, while sequential treatment showed slightly higher removals, suggesting some benefit from cavitation prior to irradiation. The final sequential mode with the addition of H_2_O_2_ was the best-performing process, but still only reached ∼ 20–25% removal. Additional H_2_O_2_ provides extra ROS generation, partially compensating for scavenging losses.

In WW matrix TMBPF and BPAF remain most reactive, consistent with TW results. BPS remained most persistent, with almost no degradation under any treatment. The relative order of reactivity was preserved, indicating that matrix effects are non-selective and impact all bisphenols similarly.

Another thing to take into account is the higher initial concentration of bisphenols in WW experiments. This matters due to competition for a limited ROS pool. In AOPs (PCO, CAV, CAV + PCO) the steady-state concentration of reactive species (^•^OH, ^•^OOH, ·O_2_–, holes and electrons) is limited. Increasing the pollutant load increases competition for those radicals and lowers the fraction of any single compound that is oxidized in a given time. A five times higher C_0_ in WW therefore consumes more ROS, leaving fewer radicals for each bisphenol and slowing apparent degradation. Also, adsorption to catalyst/film surfaces often follows nonlinear isotherms. It has been shown before that higher concentrations of BPA lead to higher adsorption [29]. This is consistent with observation in our study. WW effluent in this study also contains natural organic matter (12.63 mg/L TOC) and inorganic radical scavengers (bicarbonate, chloride). These species can consume ROS, compete with pollutants or decrease the oxidation activity of ROS [30]. The combined effect of higher pollutant concentration and background scavengers multiplies the effective competition for radicals, so the decrease in degradation percentage is larger than what *C*_0_ alone would predict.

**Fig. 5:**
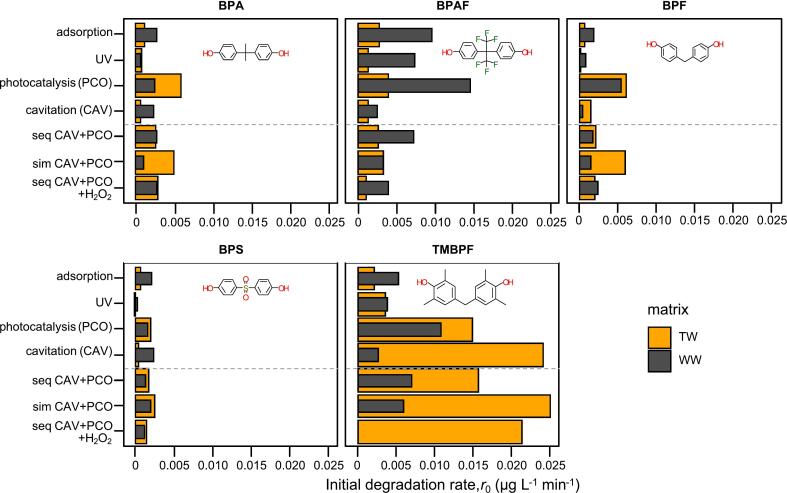
Initial degradation rates of the different regimes in the two matrices. The dashed line represents the split between the sole AOP and hybrid processes.

If true pseudo-first-order kinetics held (*r* ∝ *C*), the relative percent removal after fixed time would be independent of *C*_0_, i.e. *C*_(t)_/*C*_0_ = *e*^(−^*^k t^*^)^. More commonly, *k* decreases with increasing *C*_0_ in ROS-limited or surface-saturated regimes, so percent removal falls at higher *C*_0_. The observed lower removals in WW matrix are consistent with this behavior. For this reason we extracted the first-order reaction rate constants (*k*_app_) from the kinetic analyses of the two data sets and computed the initial degradation rates ($r_{0}$, eq. (4) with units µg/Lmin (Fig. 5a). In this way we can remove the direct dependence on *C*_0_ since *k*_app_ is a concentration-independent parameter, as well as *r*_0_, (at least in the pseudo-first-order regime) and we can isolate the intrinsic degradation efficiency of the process.

From Fig. 5 it is evident that initial degradation rates in WW as compared to TW did not fall substantially for BPA, BPAF, BPF and BPS, while they fell considerably for TMBPF. The fact that photolysis and photocatalysis showed higher *r*_0_ in WW indicates that the regime is not limited by photon absorption at these conditions. In fact, NOM can act as an inhibitor of the formation of active species via light attenuation and shielding effects while simultaneously enhancing their generation through photosensitization and electron transfer facilitation [31]. It has been reported that, dependent on the composition, NOM can act beneficial on TiO_2_′s removal efficiency [31]. In our case the effects of photosensitizing and electron transfer outweighed the negative effects of shielding and ROS quenching. This agrees with findings of Awfa et al. [32], who stated that the inhibition mechanism of NOM follows the order ROS scavenging > inner filter effect > competitive adsorption. Such behavior could thus be ascribed to photosensitizing effect of the organic compounds present in WW. Sardana et al. [33] have shown that indirect photodegradation in matrices with NOM may be improved due to hydrophobic interactions and structural links between diverse NOM polar fractions which, in turn, improve the accessibility of ROS to target pollutants.

**Fig. 6:**
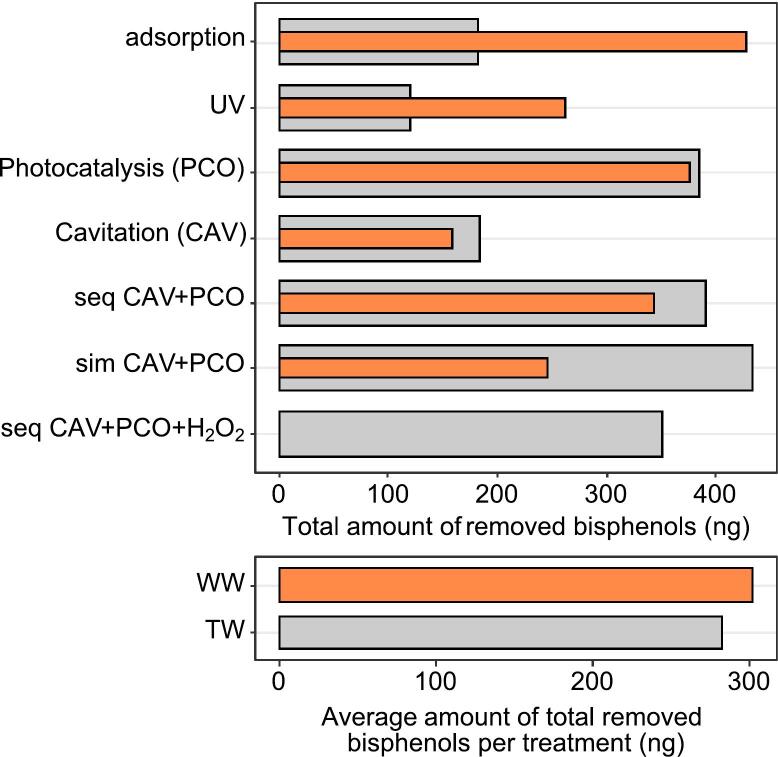
Total amounts of total bisphenols removed for specific regimes (grey bars). For sequential regime with the addition of H_2_O_2_ it was not possible to determine the total amount due to quantification issues in the WW matrix (orange columns). This regime was hence removed in the comparison calculation (bottom panel).

Since the organic load was different amongst the two matrices (Table 2), we then analysed the average removal efficiency of each process multiplied by the initial amount of injected bisphenol into the 5 L samples, which was cumulatively 1000 ng for TW and 5000 ng for WW. This way, we obtained the total amount of bisphenols removed from the solution at the end of each treatment regime (Fig. 6). It is apparent that the total amount of bisphenols removed did not vary as much as indicated in the figures of kinetics of removal (Fig. 3, Fig. 4). In fact, this result is surprising, since the added amount of bisphenols in WW treatments was five times the amount in TW experiment, i.e., 5000 ng versus 1000 ng. Such result implies on a limit of ROS production in the current system.

Additionally, increased TOC values were observed after the treatments in simultaneous and sequential configurations (Table 3). Zieliński et al. [34] have shown that HC improves the solubility of TOC in aqueous samples which may partially explain the observed data. The second reasoning could be due to the fact that the WW sample was taken after the third phase of a SBR sequence in WWTP. This stage is the aeration/reaction phase where the biomass is actively metabolizing with active extracellular enzyme activity, and ongoing production of soluble microbial products (SMPs) and extracellular polymeric substances (EPS). HC causes cell lysis and EPS disruption, releasing intracellular proteins, enzymes, nucleic acids, etc. resulting in an increase of the measured organic carbon [35], [36], [37]. However, complete understanding of this phenomenon would require future investigation.

**Table 3:** Analysis of total organic carbon (TOC) of the pristine WW and after various hybrid treatments.

**Sample**	**Organic C** **[mg/L]**	**Total C** **[mg/L]**	**Inorganic C** **[mg/L]**
Tap water	<0.3	−	−
Wastewater	12.63	85.54	72.9
CAV + PCO par.	14.74	86.53	71.79
CAV + PCO seq.	15.11	87.08	71.98
CAV + PCO + H_2_O_2_	23.82	95.84	72.01

**Fig. 7:**
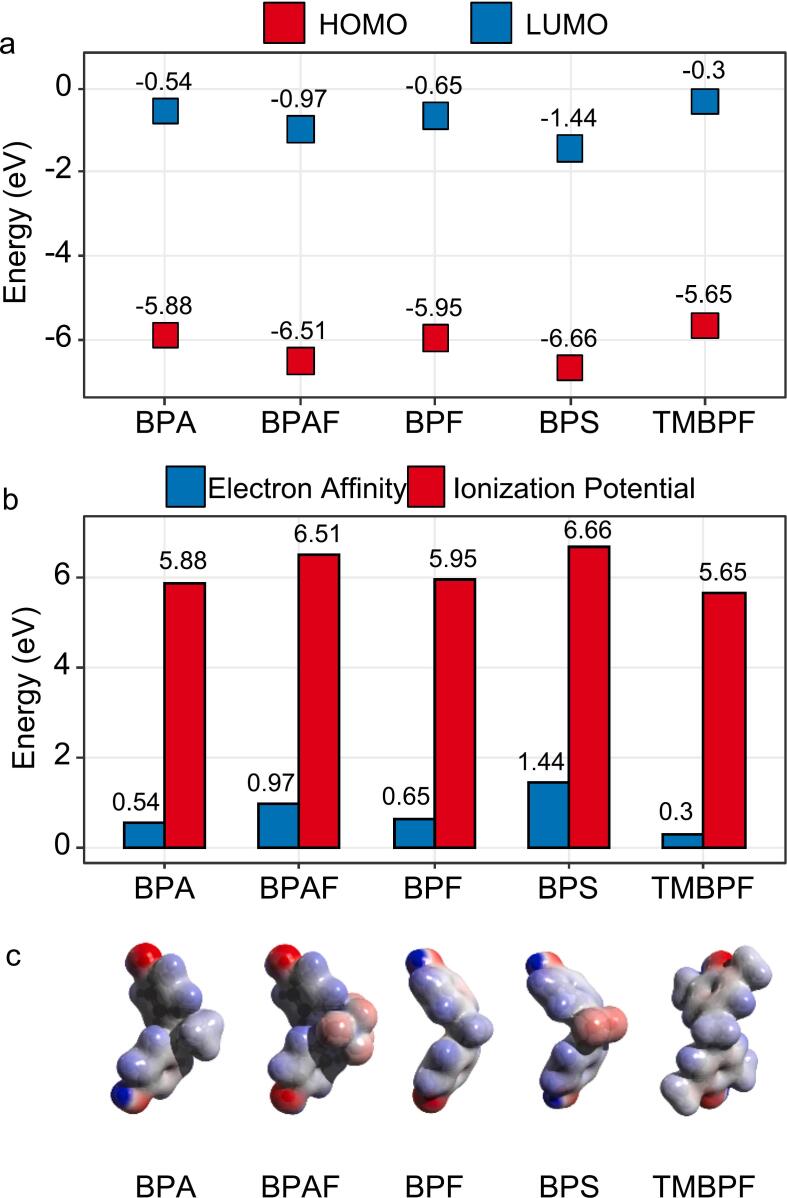
Selected molecular descriptors of the five bisphenols. (a) HOMO and LUMO positions, (b) electron affinities and ionization potentials, and (c) molecular electrostatic potential surfaces, where red and blue colours signify areas of negative and positive potential, respectively.

The susceptibility toward ·OH radical attack was assessed based on HOMO/LUMO energy, following the HSAB principle, whereby ·OH acts as a hard electrophilic radical preferentially attacking electron-rich, soft molecules [38], [39]. TMBPF exhibited the highest HOMO energy (Fig. 7), indicating the greatest susceptibility to ·OH attack, consistent with the electron-donating effect of its four methyl substituents. In contrast, BPS showed the lowest HOMO energy and the highest electron affinity (Fig. 7b), suggesting the greatest resistance to ·OH attack, attributable to the strongly electron-withdrawing sulfone bridge (Fig. 7c) [39], [40]. BPAF occupied an intermediate position, despite its electron-withdrawing C–F groups yielding a relatively low HOMO energy, its high electron affinity resulted in a narrow HOMO-LUMO gap. The computed reactivity trend (TMBPF > BPA ≥ BPF > BPAF > BPS) showed reasonable agreement with experimental data discussed above.

### 3.3 Stability of TiO_2_-SiO_2_ films

**Fig. 8:**
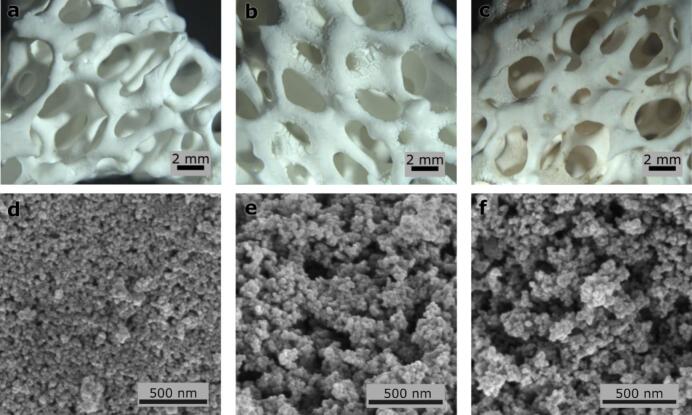
Optical images (a–c) and electronic microscope images (d–f) of pure and used catalyst films. The labels correspond to: a, d — pristine catalyst; b, e — catalyst used in TW; and c, f — catalyst used in WW.

Morphological and optical properties of the samples before and after catalytic tests were examined to confirm the suitability of the materials for their use in real life settings. The resulting films on Al_2_O_3_ layers exhibit a uniform distribution of particles (TiO_2_ and SiO_2_) with pores averaging around 15 nm (Fig. 8d–f). However, after the tests in tap water, visible cracks appeared that were around 100 µm large. Similar was the case with wastewater, with the addition of yellow colouration to the films (Fig. 8a–c). IR spectroscopy was used to identify the nature of these spots. However, the spectra showed (Supplementary file, Fig. S1) no statistical differences between the fresh or used catalysts. In contrast, EDXS analysis of the catalyst's surface showed the presence of calcium (1 wt%, Supplementary file, Fig. S2) in the spectra in the samples after the catalytic tests, which indicates contamination from the water matrix (tap and wastewater).

**Fig. 9:**
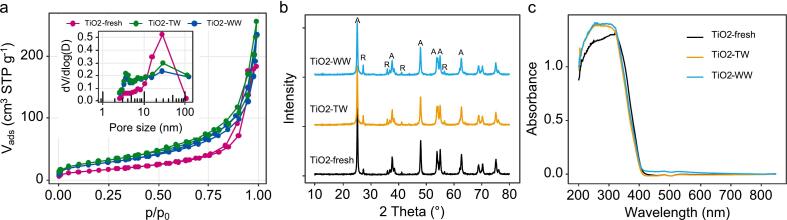
Nitrogen adsorption isotherms for the samples (a), XRD patterns (b) and solid-state UV–Vis absorption (c) before and after catalytic testing. In panel (b) the anatase and rutile phase are marked with A and R, respectively.

The shape of nitrogen sorption isotherms (Fig. 9a) is the same for all samples and is classified as Type IV according to the IUPAC classification [41] which is characteristic of mesoporous solids. This type of material is also characterised by a saturation plateau at high relative pressures (P/P_0_ = 0.95–0.99), which is observed mainly for the unused TiO_2_ sample. The hysteresis loops are of type H3 which is formed by non-rigid aggregates of plate-like particles, but also when the pore network consists of macropores that are not filled with condensate. The pore size distributions (Fig. 9a, inset) for all three samples, as determined by the BJH method, show that the number of pores of a given size decreases after the reactor tests, yet the pore structure remains the same. The two spent samples show the presence of smaller pores, i.e., the average pore diameter was 19.4, 12.9, and 14.5 nm for samples Ti-fresh, TiO2-TW, and TiO2-WW, respectively. Also, the total pore volume of the catalyst increases after the tests in the reactor system (i.e., 0.285, 0.397, and 0.361 cm^3^/g for Ti-fresh, TiO2-TW, and TiO2-WW, respectively). Such behaviour implies on the presence of additional particles/species in the pores or on the surface of the photocatalyst, present either as carbon deposits or even calcium particles from the water matrix. All of this also resulted in a significant increase in the specific surface area of the samples after the catalytic tests, where samples Ti-fresh, TiO2-TW, and TiO2-WW had values of 59, 111, and 100 m^2^/g, respectively.

The crystal structures of the samples were analysed using powder XRD. Fig. 9b shows the XRD patterns of the samples, revealing that they were polycrystalline, meaning that they contain both anatase and rutile phases, confirming the presence of P-25 photocatalyst in the films. The average size of the photocatalyst, as calculated using the Scherrer formula, did not change after the catalytic tests, as did not the optical properties of the catalysts (Fig. 9c). All samples show a band gap of ∼ 3.3 eV (376 nm), indicating their optical absorption in the UVA range.

**Fig. 10:**
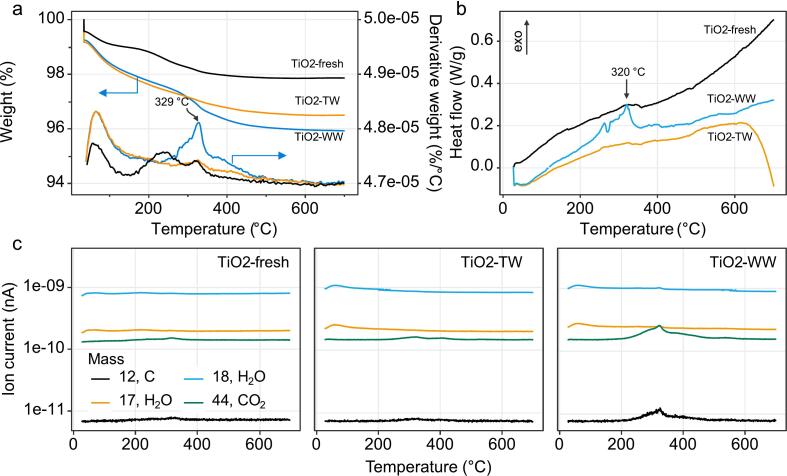
Thermal analysis of the samples. TGA (a) and DSC (b) results for the samples together with their respective dTGA curves (a, bottom). TG-MS for selected masses (c) of the three samples.

Regarding the thermal analysis of the samples (Fig. 10), the mass loss for the fresh catalyst is 2.13%, whereas for the used catalyst in tap water, the loss is 3.46%, and in wastewater, 4.05%. Both photocatalytic materials after use show increased mass loss below 120 °C which can be ascribed to presence of surface water due to their exposure to it during tests. A steep mass loss is observed in the sample TiO2-WW at ∼ 320 °C indicating on the loss of organic material in this sample. Differential scanning calorimetry (DSC) showed (Fig. 10b) an exothermic peak at 320 °C in TiO2-WW. We assume that this is an oxidative decomposition of organic matter, as thermal degradation of bisphenols occurs at much higher temperatures, i.e., higher than 450 °C [42]. The samples were further analysed by thermal analysis coupled to a mass spectrometer to confirm this hypothesis.

The CO_2_ (*m*/*z* = 44) peak occurs in all samples at around 330 °C, between 200 °C and 450 °C (Fig. 10c), following the exothermic peak described above, and is due to the decomposition of organic matter [43]. In this temperature range, smaller and larger organic molecules decompose, which is reflected in the two more pronounced exothermic peaks in the DSC curve [44]. In the case of the post-treatment sample in wastewater, the peak shape for CO_2_ is much more intense, indicating that more organic matter has decomposed. These are not only bisphenols or their oxidation products but also other unidentified organic compounds originating from the matrix, which in this case is wastewater. The second CO_2_ peak occurs at ∼ 550 °C and is due to the decomposition of carbonates. This peak is only visible in the wastewater matrix and is consistent with the calcium detected on the surface of this catalyst by XPS and SEM-EDXS.

**Fig. 11:**
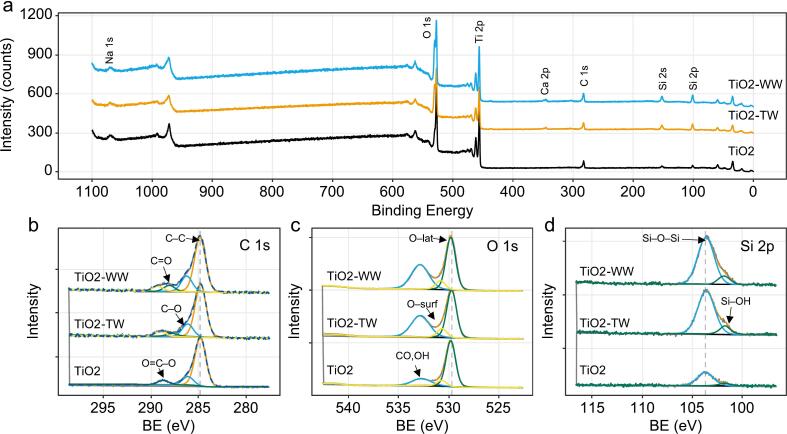
XPS analysis of the TiO_2_-SiO_2_ catalysts. Survey spectra (a), and high-resolution XPS core-level C 1 s (b), O 1 s (c) and Si 2p (d) spectra for fresh and spent catalysts.

The chemical composition and oxidation states of species present on the surface of the fresh and used TiO_2_ catalyst were investigated by XPS spectroscopy (Fig. 11). Data showed that upon using the catalysts in the reactor system, there was no change in the Ti local environment (Supplementary file, Fig. S3). Due to spin–orbit coupling, the Ti 2p spectrum was split into two peaks located at binding energies of 458.8 and 464.5 eV, which were associated with the Ti 2p_3/2_ and Ti 2p_1/2_ bands, respectively. The difference between the two bands was 5.7 eV, indicating oxidation state of titanium was Ti^4+^. This value remained unchanged after the catalytic tests.

The O 1 s spectra (Fig. 11b) of the catalysts can be deconvoluted into three peaks, namely 529.9, 530.8, and 533.0 eV, which were consistent with the presence of Ti–O, –OH, and C–O bonds, respectively [45]. An increase in the C–O band in the spent catalyst indicates the presence of organic molecules on the surface especially since the increase is the largest in the TiO_2_-WW sample. This observation is corroborated with the C 1 s core-level spectra (Fig. 11a). These contain three peaks at binding energies of 284.8, 586.3, and 588.8 eV that were attributed to adventitious carbon, C–O, and O=C–O groups, respectively [46]. Also here, an increase in the O-containing peaks is observed, more so in the TiO_2_-WW sample.

In the Si 2p spectra (Fig. 11c) the dominant Si 2p peak, denoting Si–O bond in fully oxidized SiO_2_, shifts to lower BE values from 103.70 eV in fresh catalyst to 103.62 eV when used in TW experiments, and to 103.59 eV for WW experiments (Table 4). Such a shift may reflect partial hydroxylation, adsorption of intermediates, or mild reduction in surface oxide network.

**Table 4:** Positions of the main XPS peaks for the three materials.

	**Peak Position (eV)**		
**XPS Region and Bond**	**Pristine**	**Post-tests (TW)**	**Post-tests (WW)**
O1s, O–Lat	529.78	529.72	529.76
O1s, O–Surf	530.68	530.69	530.80
O1s, CO, OH	532.71	532.84	532.83

The secondary peak at 101.75 eV remains unchanged after TW experiments. Conversely it shifts to 101.79 eV after WW regimes implying that Si–OH species are being replaced by more electron-withdrawing species (e.g. adsorbed organics, bisphenol degradation intermediates). This is due to higher complexity, ionic strength, and organic load in WW which more strongly alter the chemical environment of surface Si atoms.

## 4 Conclusions

A hybrid advanced oxidation process 5 L reactor rig was built on the basis of combining hydrodynamic cavitation (HC) and photocatalysis (PCO) techniques with flow adjusted to 6 L/min. TiO_2_ photocatalyst was successfully anchored onto Al_2_O_3_ monoliths by employing silicate binder and used in a PCO reactor directly following a HC generator. Individual HC, PCO, adsorption, photolysis and hybrid configurations were tested and compared for their efficiencies. Five bisphenols were chosen as trace environmental pollutants present at their environmentally relevant concentrations: 200 ng/L and 1000 ng/L of each.

The removal efficiencies for various bisphenols followed the trend TMBPF > BPA ≥ BPF > BPAF > BPS which could be attributed to the inverse trend of electron affinities of the molecules, as confirmed by quantum chemical calculations.

Comparing the matrix effect of tap water and wastewater, it was shown that relative kinetics of removal were severely decreased in WW but the average total amount of bisphenols removed per treatment was practically equal in both matrices. The immobilized photocatalyst was stable for up to 5 consecutive uses under the chosen experimental conditions showing applicative potential.

## CRediT authorship contribution statement

**Andraž Šuligoj:** Data curation, Formal analysis, Investigation, Methodology, Visualization, Writing – original draft, Writing – review & editing. **Mojca Zupanc:** Conceptualization, Investigation, Methodology, Project administration, Supervision, Writing – original draft, Writing – review & editing. **Jurij Gostiša:** Conceptualization, Investigation, Supervision, Validation, Writing – original draft. **Pia Hrovat:** Data curation, Investigation, Writing – original draft. **Ester Heath:** Data curation, Funding acquisition, Methodology, Supervision, Validation, Writing – review & editing. **Nataša Novak Tušar:** Conceptualization, Funding acquisition, Resources, Supervision, Writing – review & editing. **Urška Lavrenčič Štangar:** Funding acquisition, Supervision, Validation, Writing – review & editing.

## Declaration of competing interest

The authors declare that they have no known competing financial interests or personal relationships that could have appeared to influence the work reported in this paper.

## Data Availability

Data are available at the University of Ljubljana Repository at https://repozitorij.uni-lj.si/IzpisGradiva.php?id=181293.
