# Supplementary material for: Enhanced removal of bisphenols using immobilized TiO2 in photocatalytically assisted hydrodynamic cavitation
Source: Ultrason Sonochem. 2026 Mar 28;129:107830. doi: 10.1016/j.ultsonch.2026.107830 (PMC13089031; doi:10.1016/j.ultsonch.2026.107830)
Supplement: Supplementary Data 1 [file mmc1.docx]

Article

Supporting information

*for*

Enhanced Removal of Bisphenols Using Immobilized TiO_2_ in Photocatalytically Assisted Hydrodynamic Cavitation

Andraž Šuligoj ^1^, Mojca Zupanc ^2^, Jurij Gostiša ^2^, Pia Hrovat ^1^, Ester Heath ^3,4^, Nataša Novak Tušar ^5^ and Urška Lavrenčič Štangar ^1,^*

^1^ Faculty of Chemistry and Chemical Technology, University of Ljubljana, Večna pot 113, SI-1000 Ljubljana, Slovenia

^2^ Faculty of Mechanical Engineering, University of Ljubljana, Aškerčeva cesta 6, SI-1000 Ljubljana, Slovenia

^3^ Jožef Stefan Institute, Jamova cesta 39, SI-1000 Ljubljana, Slovenia

^4^ International Postgraduate School Jožef Stefan, Jamova cesta 39, SI-1000 Ljubljana, Slovenia

^5^ National Institute of Chemistry, Hajdrihova 19, SI-1000 Ljubljana, Slovenia

The parameters of the tap water used in the experiments are shown in Table S1.

**Table S1**. Parameters of tap water used in the experiments.

| **Parameter** | **Value** | **Unit** |
| --- | --- | --- |
| Temperature | 12.3 | °C |
| pH | 7.4 |  |
| El. conductivity | 451 | µS/cm |
| Cl^-^ | 7.4 | mg/L |
| NO_3_ | 13.8 | mg/L |
| SO_4_^2-^ | 9.74 | mg/L |
| HCO_3_ | 275 | mg/L |
| Calcium | 75 | mg/L |
| Magnesium | 18 | mg/L |
| Sodium | 5 | mg/L |


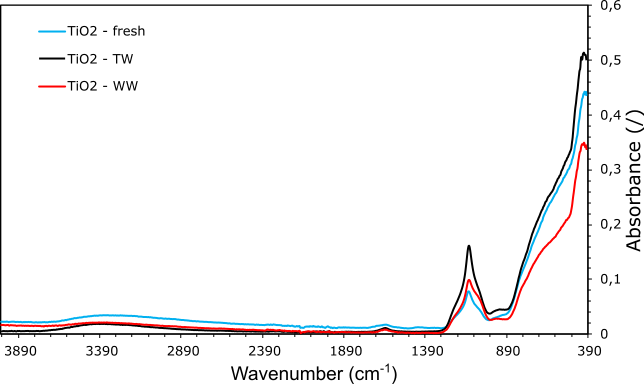


**Figure S1**. ATR spectra of the pristine and used catalysts.

**Figure S2**. SEM images and EDXS spectra for the pristine and used photocatalysts.

**Figure S3**. XPS spectra of the pristine and used samples for Ti 2p part of the spectrum
